# The Pleiotropic Antibacterial Mechanisms of Ursolic Acid against Methicillin-Resistant *Staphylococcus aureus* (MRSA)

**DOI:** 10.3390/molecules21070884

**Published:** 2016-07-07

**Authors:** Chao-Min Wang, Yun-Lian Jhan, Shang-Jie Tsai, Chang-Hung Chou

**Affiliations:** Research Center for Biodiversity, China Medical University, Taichung 40402, Taiwan; wangchaomin@mail.cmu.edu.tw (C.-M.W.); ah_giu@yahoo.com.tw (Y.-L.J.); csungjay@yahoo.com.tw (S.-J.T.)

**Keywords:** methicillin-resistant *Staphylococcus aureus* (MRSA), ursolic acid, two-dimensional gel electrophoresis (2DE)

## Abstract

(1) Background: Several triterpenoids were found to act synergistically with classes of antibiotic, indicating that plant-derived chemicals have potential to be used as therapeutics to enhance the activity of antibiotics against multidrug-resistant pathogens. However, the mode of action of triterpenoids against bacterial pathogens remains unclear. The objective of this study is to evaluate the interaction between ursolic acid against methicillin-resistant *Staphylococcus aureus* (MRSA); (2) Methods: The ability of ursolic acid to damage mammalian and bacterial membranes was examined. The proteomic response of methicillin-resistant *S. aureus* in ursolic acid treatment was investigated using two-dimensional (2D) proteomic analysis; (3) Results: Ursolic acid caused the loss of staphylococcal membrane integrity without hemolytic activity. The comparison of the protein pattern of ursolic acid–treated and normal MRSA cells revealed that ursolic acid affected a variety of proteins involved in the translation process with translational accuracy, ribonuclease and chaperon subunits, glycolysis and oxidative responses; (4) Conclusion: The mode of action of ursolic acid appears to be the influence on the integrity of the bacterial membrane initially, followed by inhibition of protein synthesis and the metabolic pathway. These findings reflect that the pleiotropic effects of ursolic acid against MRSA make it a promising antibacterial agent in pharmaceutical research.

## 1. Introduction

Pentacyclic triterpenoids, one of the most abundant natural products existing terrestrial plants, have been demonstrated to exhibit various pharmacological activities, such as hepatoprotective, anti-inflammatory, antioxidant, and anticancer effects [1,2,3,4]. The plant-derived chemicals enhancing the bacteria’s susceptibility to other antibiotics have increasingly been paid more attention. Additionally, pentacyclic triterpenoids such as betulinic acid, imberbic acid, oleanolic acid, ursolic acid and zeylasteral have also been reported to possess antimicrobial activity [5,6,7]. Moreover, ursolic acid showed a synergistic effect with ampicillin and tetracycline against both *Bacillus cereus* and *Staphylococcus aureus* [5]. The potentiality of ursolic acid in synergistic effects with antibiotics to enhance the antibacterial activity of β-lactams can constitute a valuable agent for therapeutic application [5]. However, there are limited reports involving the antibacterial mechanism on pathogenic bacteria for pentacyclic triterpenoids.

Protein expression profiling, which can be constructed by two-dimensional gel electrophoresis (2DE), is an excellent approach to demonstrate the pattern of proteins expressed; 2DE can also be applied to show to what extent each single protein is expressed in different environmental conditions. In *S. aureus* and *B. subtilis*, gel-based proteomics is also a useful approach for visualizing the responses of bacteria to stress and starvation [8]. With the advantage of mass spectrometry (MS), the 2D gel approach combined with MS for protein identification may facilitate a broad view on the physiological state of the bacterial cell, allowing us to study the cellular response of bacteria to classical antibiotics and to identify the modes of action of novel compounds [9]. Previous reports indicated that the antibacterial mechanism of the pentacyclic triterpenoids was shown to be involved in regulating the expression of genes associated with peptidoglycans and virulence factors in bacteria [10]. Therefore, the impact of ursolic acid on membrane integrity was conducted in this study. Furthermore, an attempt is also made to understand the antibacterial mechanism of ursolic acid using 2D proteomic analysis. Comparative proteome analyses were conducted to investigate whether or not the proteomic response would reveal marker proteins in ursolic acid treatment. Based on the correlation of these marker proteins with their functional properties, the antibacterial mechanisms of ursolic acid could be proposed.

## 2. Results

### 2.1. Effects of Antibiotics and Ursolic Acid on Bacterial and Mammalian Membrane Viability

**Figure 1:**
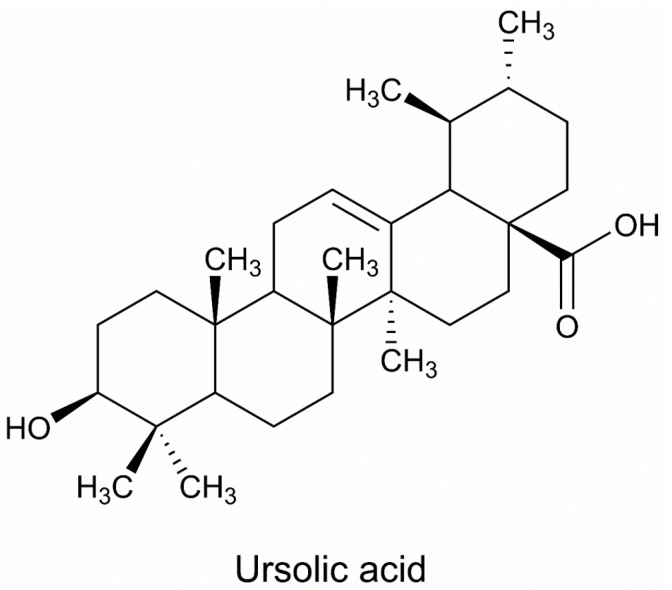
Chemical structure of ursolic acid.

Rapid bactericidal activity is a feature that common to antibacterial agents acting on bacteria by compromising the integrity of the cytoplasmic membrane. To examine whether ursolic acid acts on bacterial membranes, we employed the *Bac*Light™ assay. Previously, we have evaluated the minimum inhibitory concentration (MIC) value of antibiotics and ursolic acid (Figure 1) against methicillin-resistant *Staphylococcus aureus* (MRSA) [5]. The MIC values of ampicillin, tetracycline and ursolic acid against MRSA were 512, 8 and 64 μg/mL, respectively. MRSA were further treated with different concentrations of ursolic acid in mid-exponential phase in this study. Concentrations were chosen that reduced the growth rates to approximately 50% compared to that of the untreated control culture for membrane viability and proteomic analysis. In this study, MRSA exposed to 4× of the MIC value of ursolic acid for 60 min retained 49.5% of their membrane integrity (Table 1). MRSA exposed to 4× of the MIC value of tetracycline retained 90% of their membrane integrity, while the known membrane disrupters, such as ampicillin, significantly reduced membrane integrity over the same time period (Table 1). Both ursolic acid and antimicrobial agents caused about a 17%–19% loss in erythrocyte integrity, while the known membrane-disrupters sodium dodecyl sulfate (SDS) induced hemolysis (Table 1). The results showed that ursolic acid had little effect on the erythrocyte integrity but reduced the bacterial membrane integrity significantly (Table 1).

### 2.2. Effects of Ursolic Acid on Bacterial Protein Synthesis

**Figure 2:**
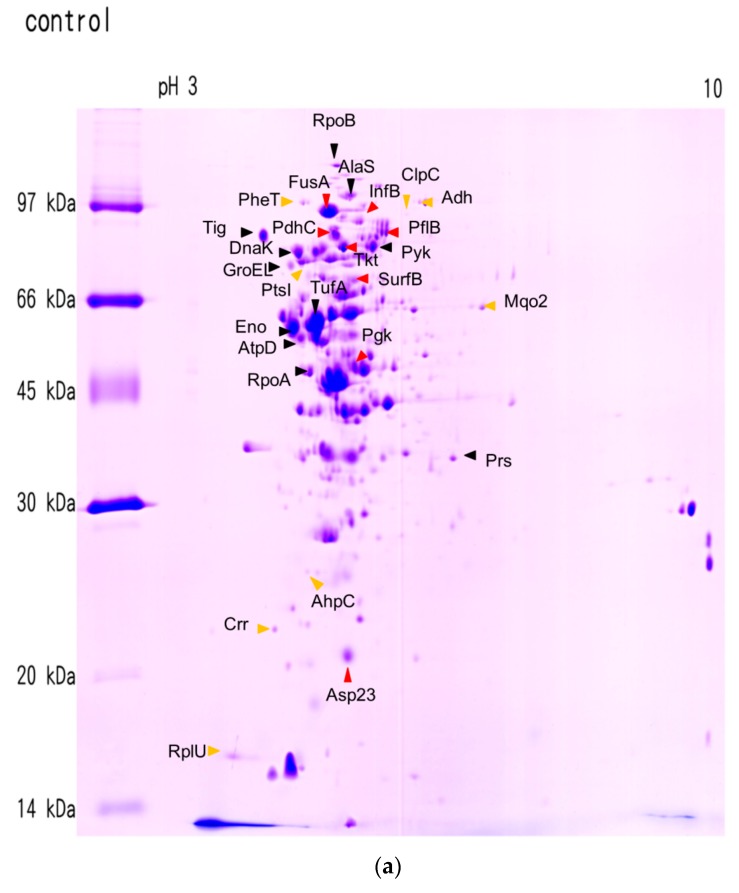

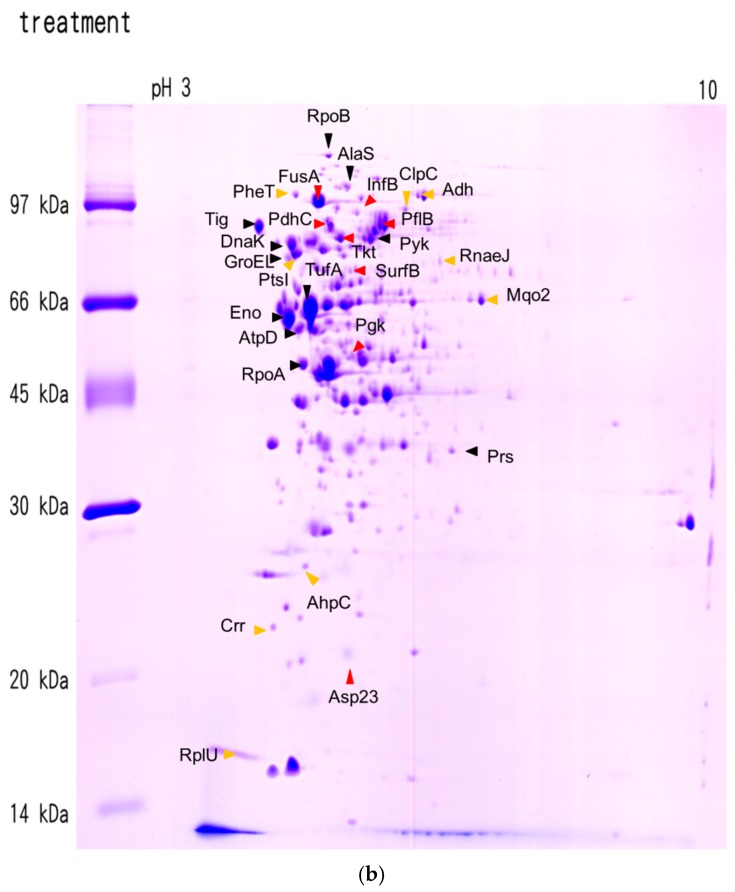
The 2D gel showing spots of interest with proteins isolated from untreated MRSA (**a**) and proteins from ursolic acid–treated MRSA (**b**); 2D-PAGE was used to separate cytoplasmic proteins in the *p*I range of three to 10 according to their *p*Is and molecular weight. Proteins induced by the ursolic acid appear yellow, repressed proteins appear red, and proteins with no significantly changes are indicated as black.

Crude proteins from MRSA cultures were extracted and subjected to 2D PAGE for the separation and analysis of protein spots. Approximately 271 protein spots were identified in the control group and 319 spots in the treatment group, ranging in *p*I between 4.0 and 10.0, and in molecular weight between 10 and 150 kDa (Figure 2). By matching both images obtained from the control and treatment groups, 200 protein spots were identified in each group. Proteins induced or reduced at least 1.5-fold are referred to as marker proteins. They proved the information about the antibiotic mode of action and reflected the physiological stress conditions of bacteria. Using MALDI-TOF/TOF, 29 protein spots were identified, ranging in *p*I between 4.3 and 9.7, and in molecular weight between 11 and 133 kDa (Table 2). Changes in protein intensity were statistically evaluated by employing the control value without ursolic acid as the baseline. The protein spots that changed in intensity significantly under ursolic acid treatment are listed in Table 3 and the position of the corresponding protein spots on the MRSA 2D gel is shown in Figure 2. Interestingly, the treatment of MRSA with ursolic acid leads to an induction of phenylalanyl-tRNA synthase and ribosomal protein L21 (Table 2) and a shift in the *p*I value of ribosomal protein L21 (Figure 1). The elongation factor G and translation initiation factor IF-2 were detected as decreased in this study. No significant change was detected in the production of the transcriptional protein RNA polymerase α and β chain. The most notable changes in the protein expression by 2D proteomic analysis indicated that ursolic acid had a marked effect on the expression of important MRSA proteins in folding and degrading the system, such as ClpC and trigger factor and chaperon proteins. The expression of enzymes involved in glycolysis was found to be decreased, but those involved in the tricarboxylic acid (TCA) cycle showed varying patterns of expression (Table 3). Essentially, glycolysis was repressed by the decreased concentration of Pgk, PdhC and Tkt proteins. The phosphotransferase system was induced by increasing the concentration of PtsI and Crr proteins. Proteins involved in the oxidative response (AhpC) and alcohol dehydrogenase (Adh) were also induced via ursolic acid treatment. Other proteins including the Fe-S cluster assembly protein (SufB), ribose-phosphate pyrophosphokinase (Prs), alkaline shock protein 23 (Asp23) and glyoxal reductase (AKRs) were repressed in this study. Although these uncorrelated proteins do not showed rational insights into the antibacterial mechanism, they still serve as specific markers, as they are reproducibly reduced after ursolic acid treatment.

**Figure 3:**
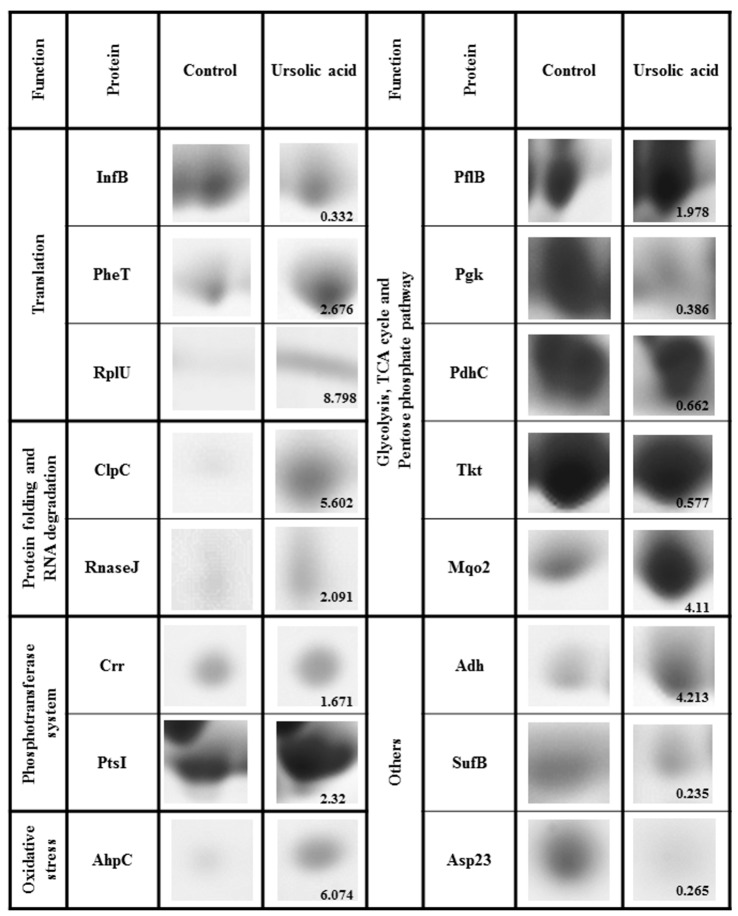
Details of 2D gels depicting 16 specific marker proteins for cell translation, protein folding and RNA degradation, the cell phosphotransferase system, oxidative stress, and the general cell metabolic pathway. Induction or reduction factor is presented in the lower right corner as the average over three replicates.

As shown in Figure 3, details of 2D gels depicting 16 specific marker proteins were presented. Proteins induced or reduced at least 1.5-fold are referred to as marker proteins. In response to translational interpretation, translation initiation factor IF-2 (InfB) was reduced while the phenylalanyl-tRNA synthase subunit beta (PheT) and 50S ribosomal protein L21 (RplU) were induced. In addition, the protein folding and degrading system (ClpC, RnaseJ), the bacterial phosphoenolpyruvate sugar phosphotransferase system (PtsI and Crr proteins), and the oxidative response (Ahp C) were all induced in ursolic acid treatment.

**Figure 4:**
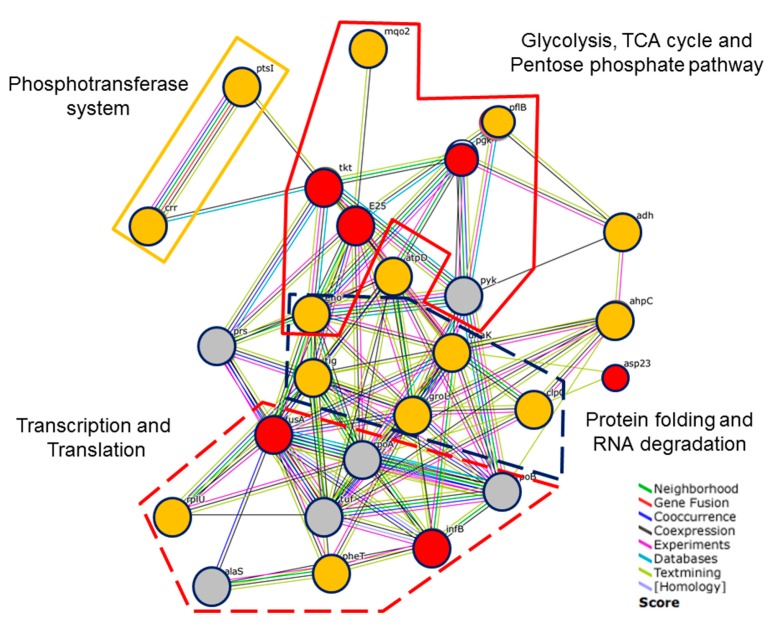
Differentially expressed proteins of ursolic acid–treated MRSA compared with controls. Dots in different colors represent repressed (red), induced (yellow) or no change (gray) in the presence of ursolic acid by quantitative proteomics. The identified proteins were classified by the annotation system Database for Annotation, Visualization and Integrated Discovery (DAVID) (version 6.7). The lines represent putative protein interactions recorded or predicted by STRING (version 10.0).

The identified proteins were classified by the annotation system of the Database for Annotation, Visualization and Integrated Discovery (DAVID) (version 6.7) [11]. Proteins were classified as four major pathways including transcription and translation, RNA degradation, the phosphotransferase system and glycolysis and the pentose phosphate pathway. In order to find relevant proteins among the multiple proteins obtained by proteomic analysis, 26 different proteins from Table 2 were subjected to the STRING database [12] for bioinformatics analysis. The STRING database integrates interaction data from several bioinformatics sources and provides information about physical and functional properties of known and predicted interactions of genes and proteins. As shown in Figure 4, each node represents a protein, and each edge represents an interaction. By mapping the affected proteins to central pathways, a number of proteins connected with each other in different metabolism pathways were found to be altered in MRSA by exposure to ursolic acid (Figure 4). The response to translational interpretation appeared to concentrate the cell’s biosynthetic activities on the protein folding and degrading system (DnaK, GroEL, Eno) at the expense of metabolic functions (PflB, Pgk, PdhC and Tkt). In addition, those responses also triggered the induction of PtsI and Crr protein, the bacterial phosphoenolpyruvate sugar phosphotransferase system (PTS), to mediate the uptake and phosphorylation of carbohydrates and control metabolism in response to carbohydrate availability. The connection of AhpC with DnaK, ClpC and GroEL indicated that proteins involved in the oxidative response may also trigger the protein folding and degrading system.

## 3. Discussion

### 3.1. Mode of Action I: Membrane Disruption

Studies on the antimicrobial mechanisms of oleanolic acid and ursolic acid demonstrated that both of the pentacyclic triterpenoids can modulate resistance to two β-lactam antibiotics, ampicillin and oxacillin, in four bacterial pathogens [13]. Two 6-oxophenolic triterpenoids, zeylasteral and demethylzeylasteral, which were isolated from the root of *Maytenus blepharodes*, have antimicrobial activity against *Bacillus subtilis* [6]. Those triterpenoids block cell division by inhibiting DNA synthesis and macromolecular synthesis in *Bacillus subtilis*. In addition, the sesquiterpene farnesol can inhibit the recycling of the lipid carrier of the murein monomer precursor and can also reduce the secretion and activity of β-lactamase, thus contributing to the increased susceptibility to β-lactams in methicillin-resistant *S. aureus* [14]. In this study, the mode of action of ursolic acid appears to be the influence on the integrity of the bacterial membrane initially. The fact that ursolic acid acts to compromise the integrity of the bacterial membrane may explain why this compound has the synergistic ability to increase the antibacterial activity of antibiotics. In addition, there is evidence to suggest that bacteria are not resistant to membrane-active agents [15,16]. Thus, the membrane-perturbing ability of ursolic acid is also an advantage in restricting the selection of bacterial resistance.

### 3.2. Mode of Action II: Translation Interruption

A previous study indicated that both the transcriptional inhibitor and the various translational inhibitors all triggered an increased relative rate of synthesis of the components of the transcriptional and the translational machinery (RNA polymerase, elongation factors, ribosomal proteins, tRNA synthetases) in *Haemophilus influenzae* [10] and *B. subtilis* [9]. However, ursolic acid triggered a different way of decreasing the relative concentration of the elongation factor and *p*I changing in ribosomal proteins in this study. This implies that the signal for the regulation of RNA polymerase constituents as well as for ribosome subunits lies in the impairment of protein synthesis, regardless of whether it takes place at the level of mRNA synthesis or in ribosome function. The accumulation of mRNA and misfolded proteins leads to an induction of ribonuclease and chaperon subunits. Additionally, the Clp protein, comprising an ATPase specificity factor and a proteolytic domain, has been demonstrated to play a role in bacterial adaptation to multiple stresses by the degradation of accumulated misfolded proteins. In *S. aureus*, ClpC has been shown to regulate the TCA cycle, growth in recovery from the stationary phase and cell death, the oxidative stress response, autolysis, and DNA repair [17,18]. ClpC is a likely sensor of stress encountered during both environmental stress and infection [19]. Thus, ursolic acid may act as a protein synthesis inhibitor that interferes with translation accuracy–induced Clp proteins known to be induced by misfolded proteins.

### 3.3. Mode of Action III: Metabolic Pathway Interaction

Moreover, most of the metabolic enzymes involved in glycolysis displayed a decrease in their relative rate of synthesis upon treatment with ursolic acid. The response to protein synthesis inhibition appears to concentrate the cell’s biosynthetic activities on the protein folding and degrading system at the expense of metabolic functions. Those responses may also trigger the induction of PtsI and Crr protein and the bacterial phosphoenolpyruvate sugar phosphotransferase system (PTS), to mediate the uptake and phosphorylation of carbohydrates and to control the metabolism in response to carbohydrate availability. Furthermore, alcohol dehydrogenase (Adh) is induced via NAD^+^ accumulation, which is a result caused by glycolysis repression. Glycolysis is an important process of ATP generation via the substrate level of phosphorylation. The breakdown of glucose generates more ATP in oxidative phosphorylation while the same monosaccharides produce only two net ATP in glycolysis [20]. During the repression of glycolysis, bacteria accumulated phosphoenol pyruvate (PEP) via enolase (Eno) and malate quinone oxidoreductase 2 (Mqo2) inductions. The formation of phosphoenol pyruvate (PEP) enables the synthesis of more ATP during oxidative phosphorylation.

### 3.4. Mode of Action IV: Oxidative Stress Response

Alkyl hydroperoxide reductase subunit C (AhpC) is the catalytic subunit responsible for the detoxification of reactive oxygen and facilitates the survival of pathogenic bacteria under environmental stress or during infection [21]. In *Salmonella typhimurium*, AhpC protects bacterial cells against reactive nitrogen intermediates [22]. Recently, a similar publication concluded that antibiotics pose an oxidative stress on the cells, and combating this stress is a general antibiotic response [23]. Ursolic acid may also elicit the oxidative response in MRSA via the AhpC induction.

**Figure 5:**
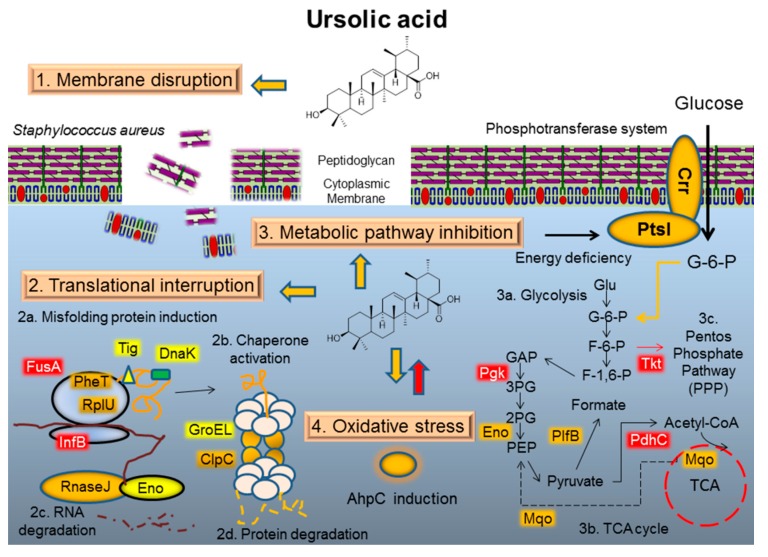
The hypothetical mechanism of ursolic acid against MRSA. Initially, ursolic acid contacts MRSA and causes a destructive effect on the bacterial membrane. Later on, ursolic acid affects the proteins that are involved in the translation process with translational accuracy. The accumulation of mRNA and misfolded protein leads to an induction of ribonuclease and chaperon subunits. Furthermore, most of the metabolic enzymes involved in glycolysis display a decrease in their relative rate of synthesis upon treatment with ursolic acid. The repression of glycolysis and the pentose phosphate pathway induces the production of PtsI and Crr protein, the bacterial phosphotransferase system (PTS), to mediate the uptake and phosphorylation of carbohydrates and to control the metabolism in response to energy deficiency. Finally, ursolic acid elicits the oxidative response in MRSA. AhpC is induced in order to protect bacterial cells against reactive ursolic acid.

As shown in Figure 5, the mechanism of ursolic acid against MRSA was illustrated. Initially, ursolic acid contacts MRSA and causes a destructive effect on the bacterial membrane. Later on, ursolic acid affects the proteins that are involved in the translation process with translational accuracy. The accumulation of mRNA and misfolded protein leads to an induction of ribonuclease and chaperon subunits. Furthermore, most of the metabolic enzymes involved in glycolysis display a decrease in their relative rate of synthesis upon treatment with ursolic acid. The repression of glycolysis and the pentose phosphate pathway induces the production of PtsI and Crr protein, the bacterial phosphotransferase system (PTS), to mediate the uptake and phosphorylation of carbohydrates and to control the metabolism in response to energy deficiency. Finally, ursolic acid elicits the oxidative response in MRSA. AhpC is induced in order to protect bacterial cells against reactive ursolic acid.

## 4. Materials and Methods

### 4.1. Effects of Antimicrobial Agents on Bacterial and Mammalian Membranes Viability

The effect of antibacterial compounds on the integrity of the staphylococcal membrane after one hour exposure was assessed using the *Bac*Light™ assay according to the report previously [16]. Briefly, MRSA was exposed to a concentration of antibacterial agent equivalent to 4 × MIC for 60 min at 37 °C. Mixture was centrifuged at 3000 g for 5 min and resuspended in PBS buffer. The MRSA was adjusted suspensions to 1 × 10^7^ cfu/mL (~0.15 OD_670_), mixed with reagents and incubated for 15 min in dark. Integrated intensities of the green (510–540 nm) and red (620–650 nm) emissions were acquired by fluorescence microplate reader. Bacteria with intact cell membranes stain fluorescent green, whereas bacteria with damaged membranes stain fluorescent red. The ratio of green to red fluorescence emission is proportional to the relative numbers of live bacteria and bacterial membranes viability.

The ability of compounds to damage mammalian membranes was examined by measuring the hemolysis of sheep erythrocytes [24]. Fresh whole sheep blood was treated with heparin, centrifuged at 1000 g for 10 min at 4 °C, and discarded the supernatant. The erythrocyte pellet was washed and resuspended to 5% *v*/*v* in 10 mM Tris-HCl buffer containing 0.85% NaCl. Erythrocytes were further diluted 10-fold in buffer, and exposed to a concentration of antibacterial agent equivalent to 4 × MIC for MRSA for 1 h at 37 °C. The hemolysis of sheep erythrocytes was measured at OD_540_ and the addition of 5% (*w*/*v*) sodium dodecyl sulfate (SDS) was defined as 100% hemolysis.

### 4.2. 2D Gel Electrophoresis Analysis

The bacteria were treated with ursolic acid (Sigma, St. Louis, MO, USA) at a concentration corresponding to 4 × MIC for 60 min according to previous reports [9,16]. This concentration decreases the growth rate of a mid-log-phase culture by 50% (Table 3) and could therefore be expected to trigger major changes in protein expression. Two-dimensional gel electrophoresis was performed by using the Ettan™ IPGphor II Isoelectric Focusing system (GE Healthcare, Piscataway, NJ, USA) according to the instructions of the manufacturer. Protein samples (300 μg) were separated by using IPG strips in pH range of 3–10. Isoelectric focusing was performed by using 7 M urea, 2 M thiourea, 4% CHAPS and 40 mM DTT with increasing voltage. Rod gels were soaked for 15 min at ambient temperature in equilibration buffer (50 mM Tris-HCl, pH 8.8, 6 M urea, 30% glycerol, 2% SDS, 10 mg/mL DTT) and applied to a second dimension using a commercially available 12% Tris-Glycine SDS gel (13 cm × 13 cm × 1.0 mm) (Pharmacia, Uppsala, Sweden) with an Ettan™ DALTsix Large Vertical System (GE Healthcare, Uppsala, Sweden). Image was scanned by ImageScanner™ (GE Healthcare, Uppsala, Sweden) and analyzed by ImageMaster™ 2D Platinum (GE Healthcare, Uppsala, Sweden).

### 4.3. In-Gel-Preparation of Tryptic Peptides

Coomassie-stained protein bands were excised from the gel and washed three times for 10 min with water. Reduction was performed with 100 mM DTT/50 mM NH_4_HCO_3_ for 30 min at 56 °C, and the proteins were subsequently alkylated with 55 mM iodoacetamide in the dark for an additional 20 mins at room temperature. Gel pieces were equilibrated twice with 1 mL of 50 mM NH_4_HCO_3_ (pH 7.8) for 10 min, shrunk with 1 mL of acetonitrile, rehydrated with 1 mL of 50 mM NH_4_HCO_3_ and finally shrunk again with acetonitrile. After air-drying, gel pieces were reswollen in a digestion buffer, containing 50 μL of 50 mM NH_4_HCO_3_, and 0.05 mg of trypsin (Promega, Madison, WI, USA) at 37 °C for 16 h. Peptides were extracted by subsequent incubation for 30 min at room temperature with 50 mM NH_4_HCO_3_, 0.1% TFA and finally 0.1% TFA/acetonitrile (1:2 *v*/*v*).

### 4.4. LC-MS/MS Analysis

LC-MS/MS analysis was performed on an integrated nanoLC-MS/MS system (QSTAR XL, AB SCIEX, Framingham, MA, USA) comprising a LC Packings NanoLC system with an autosampler, and a QSTAR XL Q-Tof mass spectrometer (AB SCIEX, Framingham, MA, USA) fitted with nano-LC sprayer. Injected samples were first trapped and desalted on a LC-Packings PepMap™ C18 μ-Precolumn™ Cartidge (5 μm, 30 μm inner diameter × 5mm, ThermoFisher Scientific Inc., Waltham, MA, USA). Peptides were separated on an analytical LC-Packings PepMap C18column (3μm, 75 mm inner diameter × 15 cm, ThermoFisher Scientific Inc.) connected inline to the mass spectrometer, using a 45 min gradient of 5% to 60% acetonitrile in 0.1% formic acid. Data of MS/MS were fully automated and synchronized with AnalystQS (version 1.0) software. For protein identification analysis, the one second survey scans were acquired over the mass range *m*/*z* 400–1600 and a maximum of 10 concurrent MS/MS acquisitions could be triggered for 2+, 3+ and 4+ charged precursors detected at an intensity above the predefined threshold. The peak list files were used to query the NCBI database using the Mascot for web search (Matrix Science, Boston, MA, USA).

### 4.5. Data Analysis

The annotation system, Database for Annotation, Visualization and Integrated Discovery (DAVID) (version 6.7) [11], was used for analysis of differentially expressed proteins by Gene Ontology assignment. Identified proteins were uploaded into the DAVID functional annotation tool and compared to the proteome background of *S. aureus*. The relationship between differentially expressed proteins in ursolic acid treated MRSA and their interaction with other proteins were analyzed using STRING database 10.0 [12]. All statistical analyses were conducted using SPSS 13.0. All results have been expressed as average mean ± standard deviation (S.D.) values. ANOVA was used to evaluate differences and a *p* value of <0.05 was considered significantly. Turkey’s multiple range test was also used to evaluate the means. Different letters represent values that were significantly different at α = 0.05 level.

## 5. Conclusions

In this study, the mode of action of ursolic acid appears to be the influence on the integrity of the bacterial membrane initially, followed by the inhibition of protein synthesis and the metabolic pathway. These results suggest that the proteomics approach gives new insights into the bacterial response toward antibacterial compounds with unknown mechanisms of action, which should prove useful in the process of antibiotic drug discovery. The pleiotropic effects of ursolic acid against MRSA make it a promising antibacterial agent in pharmaceutical research.

## Figures and Tables

**Table 1:** Effects of ursolic acid and antibiotics at 4 folds of the MIC on MRSA bacterial membrane.

Antimicrobial Compounds	Bacterial Membrane Integrity (%)	Erythrocyte Integrity (%)
None	99.4 ± 7.5 ^a^	101.8 ± 2.9 ^a^
5% SDS	0 ± 0 ^d^	0 ± 0 ^c^
Ampicillin	17.9 ± 0.6 ^c^	81.0 ± 2.6 ^b^
Tetracycline	87.3 ± 9.6 ^a^	82.2 ± 2.3 ^b^
Ursolic acid	49.5 ± 0.8 ^b^	83.0 ± 5.0 ^b^

Different letters represent values that were significantly different at α = 0.05 level.

**Table 2:** Proteins of MRSA identified by LC-MS/MS fingerprints in treatment of ursolic acid.

Protein	Protein Name	Accession No.	*p*I	M.W.	Coverage (%)
Adh	Alcohol dehydrogenase	gi|487362910	5.63	94,886	30%
AhpC	Alkyl hydroperoxide reductase subunit C	gi|445974926	5.06	20,846	38%
AKRs	glyoxal reductase	gi|446374225	5.09	31,261	14%
AlaS	Alanyl-tRNA synthase	gi|446656721	5.00	98,479	13%
Asp23	alkaline shock protein 23	gi|446137381	5.27	19,060	27%
AtpD	ATP synthase subunit beta	gi|446433275	4.71	51,382	40%
ClpC	ATP-dependent Clp protease, ATP-binding subunit ClpC	gi|446819870	5.51	90,968	11%
Crr	Glucose-specific phosphotransferase enzyme IIA	gi|261278560	4.64	63,097	15%
DnaK	Chaperone protein DnaK	gi|445956852	4.70	66,319	40%
Eno	Enolase	gi|447044501	4.58	47,115	43%
FusA	Translation elongation factor G	gi|395759323	4.80	76,530	37%
GroEL	Chaperonin protein, 60 kDa	gi|657020658	4.59	57,537	16%
InfB	Translation initiation factor IF-2	gi|445965771	5.09	77,795	18%
Mqo2	Malate quinone oxidoreductase 2	gi|447052792	6.12	55,978	13%
PdhC	Dihydrolipoyllysine-residue acetyltransferase component of pyruvate dehydrogenase complex	gi|2499415	4.87	46,411	17%
PflB	Formate acetyltransferase	gi|446817402	5.31	84,822	24%
Pgk	Phosphoglycerate kinase	gi|446997500	5.17	42,603	42%
PheT	phenylalanyl-tRNA synthase subunit beta	gi|446831715	4.71	88,838	19%
Prs	Ribose-phosphate pyrophosphokinase	gi|446856516	5.88	35,292	11%
PtsI	Phosphoenolpyruvate protein phosphotransferase	gi|446696933	4.82	34,929	11%
Pyk	Pyruvate kinase	gi|447155392	5.23	63,063	47%
RnaseJ	Ribonuclease J2	gi|445974731	5.81	62,591	18%
RplU	50S ribosomal protein L21	gi|75530481	9.78	11,309	50%
RpoA	RNA polymerase, α chain	gi|686416814	4.66	34,947	24%
RpoB	RNA polymerase, β chain	gi|686122810	4.91	133,152	19%
SufB	Fe-S cluster assembly protein	gi|446997144	5.08	52,498	19%
Tig	Trigger factor	gi|446049710	4.34	48,577	27%
Tkt	Transketolase	gi|446403587	5.00	72,212	34%
TufA	Translation elongation factor Tu	gi|446963310	4.77	43,077	56%

**Table 3:** Changes in protein expression in MRSA following treatment with ursolic acid.

Protein	Protein Name	Ratio ^1^ Treatment/Control
Transcription		
RpoB	RNA polymerase, β chain	1.042
RpoA	RNA polymerase, α chain	1.123
Translation		
FusA	Translation elongation factor G	0.685
TufA	Translation elongation factor Tu	0.958
InfB	Translation initiation factor IF-2	0.332
AlaS	Alanyl-tRNA synthase	0.975
PheT	phenylalanyl-tRNA synthase subunit beta	2.676
RplU	50S ribosomal protein L21	8.798
Protein folding and RNA degradation		
ClpC	ATP-dependent Clp protease, subunit ClpC	5.602
GroEL	Chaperonin protein, 60 kDa	1.387
Tig	Trigger factor	1.368
DnaK	Chaperone protein DnaK	1.282
Eno	Enolase	1.387
RnaseJ	Ribonuclease J2	2.091
Glycolysis, TCA cycle and Pentose phosphate pathway		
PflB	Formate acetyltransferase	1.978
Pgk	Phosphoglycerate kinase	0.386
PdhC	Dihydrolipoyllysine-residue acetyltransferase component of pyruvate dehydrogenase complex	0.662
Pyk	Pyruvate kinase	1.118
Tkt	Transketolase	0.577
Mqo2	Malate quinone oxidoreductase 2	4.11
Phosphotransferase system		
PtsI	Phosphoenolpyruvate-protein phosphotransferase	2.32
Crr	Glucose-specific phosphotransferase enzyme IIA	1.671
Oxidative stress		
AhpC	Alkyl hydroperoxide reductase subunit C	6.074
Others		
Adh	Alcohol dehydrogenase	4.213
SufB	Fe-S cluster assembly protein	0.235
AKRs	Glyoxal reductase	0.708
Prs	Ribose-phosphate pyrophosphokinase	0.869
AtpD	ATP synthase subunit beta	1.473
Asp23	alkaline shock protein 23	0.265

^1^ Ratio is defined as the % volume of treatment/% volume of control. The value >1.5 or <0.67 is recognized as a positive or negative regulation.
